# Sucrose Solution Ingestion Exacerbates Dinitrofluorobenzene-Induced Allergic Contact Dermatitis in Rats

**DOI:** 10.3390/nu16121962

**Published:** 2024-06-20

**Authors:** Aya Fujii, Ryuto Kimura, Azumi Mori, Yukihiro Yoshimura

**Affiliations:** Department of Nutrition, Kobe Gakuin University, 518 Arise, Ikawadani-cho, Nishi-ku, Kobe City 651-2180, Japan

**Keywords:** sucrose, allergic contact dermatitis, short-chain fatty acids, sodium

## Abstract

Allergic dermatitis is a skin disease with growing prevalence worldwide that has been associated with diets high in fats and sugars. Regular consumption of sucrose-containing beverages may increase the risk for several health problems, including allergic diseases and particularly asthma, but the association between sucrose consumption and allergic dermatitis is understudied. We investigated the effects of sucrose solution intake on allergic contact dermatitis in rats and found early exacerbation of 2,4-dinitrofluorobenzene (DNFB)-induced disease symptoms and altered composition of the gut microbiota after 14 d of intake. The levels of short-chain fatty acids—produced by fermentation by the intestinal microbiota—were not affected in the cecal contents and feces but decreased in the blood; this effect was especially notable for acetate. To restore blood acetate concentrations, triacetin was mixed with a 10% sucrose solution and fed to the rat model. This strategy prevented the early exacerbation of DNFB-induced symptoms. The decreased absorption of short-chain fatty acids from the intestinal lumen was not linked to the decreased expression of short-chain fatty acid transporters in the small intestine; instead, the mechanism involves a reduction in the sodium concentration in the intestinal lumen due to increased expression of sodium–glucose transporter 1 (SGLT1).

## 1. Introduction

Recent studies have shown that habitual consumption of sucrose has adverse effects on health, causing obesity and lifestyle-related diseases. The 2005 World Health Organization (WHO) guideline “Sugar Intakes for Adults and Children” estimated that sugar intake exceeding 10% of the total energy intake increases the incidence of non-communicable diseases such as dental caries and obesity. According to a 2010 study, 180,000 deaths worldwide were attributed to the consumption of sugar-sweetened beverages [1]. Studies conducted thus far have indicated that the consumption of sugar-sweetened beverages is decreasing in many developed countries. Despite this trend, consumption levels remain high, and, in many developing countries, intake continues to increase [2,3,4,5,6,7]. A 2018 cross-sectional survey of Chinese elementary and middle school students found that sugar-sweetened beverages accounted for 10–15% of the total calories consumed by elementary school students [8]. Studies in Malaysia have shown a sharp rise in the consumption of processed foods and sugar-sweetened beverages owing to rapid urbanization and economic growth. This shift has contributed to a higher prevalence of non-communicable diseases such as obesity and diabetes [9]. As previously mentioned, the increasing consumption of sugar-sweetened beverages in many countries has become a health concern in recent years [10,11].

Excessive sucrose intake has adverse effects on various inflammatory conditions [12]. A dietary intake of 50% sucrose exacerbates dextran sulfate sodium (DSS)-induced colitis [13]; the Western diet increases susceptibility to imiquimod-induced psoriasis and worsens symptoms [14]; and a dietary intake of approximately 45% sucrose exacerbates DSS-induced colitis and causes mild systemic inflammation [15]. Recently, it was reported that the disease symptoms of allergic contact dermatitis (ACD) induced by oxazolone were exacerbated in mice fed ad libitum condensed milk containing 68% sucrose in addition to the standard diet, resulting in epidermal thickening. The underlying mechanism is believed to be the high-sucrose diet, which negatively affects the levels of liver cholesterol and triglyceride (TG) [16]. However, the mechanism by which sucrose intake aggravates the symptoms of dermatitis remains unclear.

This study aimed to elucidate the effects of consuming a 10% (*w*/*v*) sucrose solution, which is hypothesized to represent the sugar-sweetened beverages commonly consumed by humans. We investigated the effects of sucrose solution intake on 2,4-dinitrofluorobenzene (DNFB)-induced ACD to provide mechanistic insights. We found that sucrose intake increased glucose and sodium absorption by upregulating the expression of sodium–glucose transporter 1 (*SGLT1*) in the small intestine. This negatively affected the concentration of sodium in the gastrointestinal lumen and, in turn, suppressed the absorption of short-chain fatty acids (SCFAs), particularly acetate, in the intestinal tract and exacerbated ACD symptoms. We suggest that excessive sucrose intake may exacerbate ACD symptoms by reducing the absorption of SCFAs from the intestinal tract.

## 2. Materials and Methods

### 2.1. Rats, Experimental Diets, and Sucrose Solution

**Figure 1:**
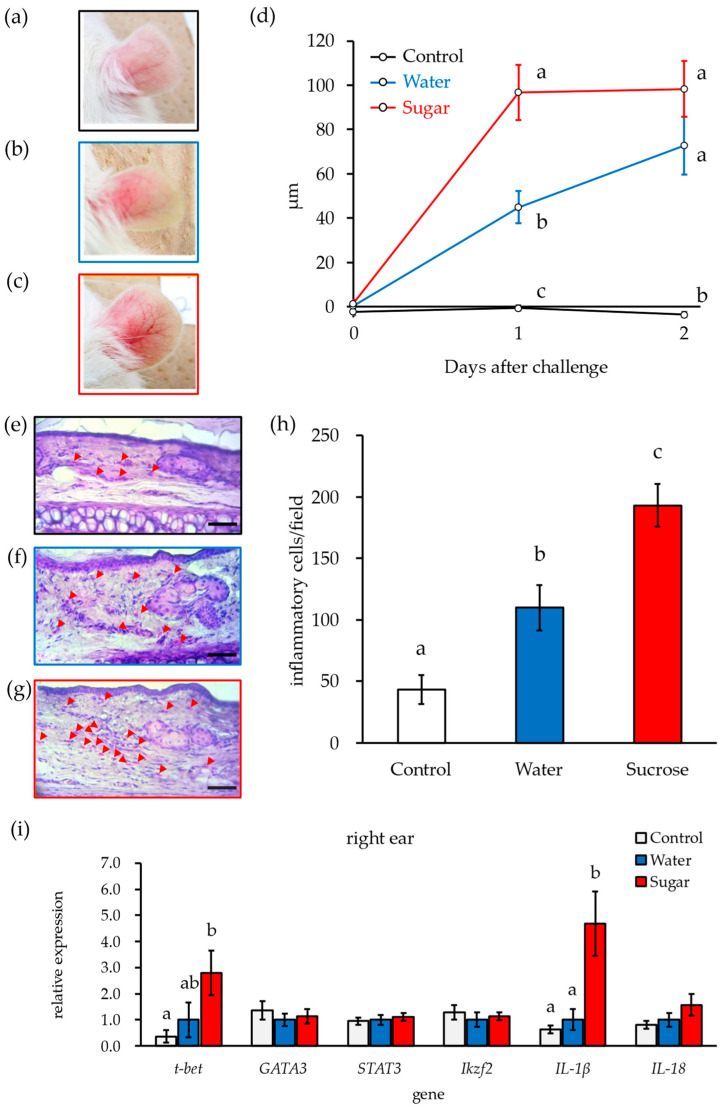
The ingestion of a 10% sucrose solution accelerates the early deterioration of DNFB-ACD symptoms. (**a**–**c**) Photographs of the right auricle allergically sensitized by DNFB: (**a**) control group, (**b**) Water group, (**c**) Sucrose group. (**d**) Time course of thickening of the right auricle sensitized to DNFB. (**e**,**f**) HE staining of tissue sections of the right auricle: (**e**) control group, (**f**) Water group, (**g**) Sucrose group. Red arrows indicate granulocytes; the scale bar indicates 50 µm. (**h**) Number of granulocytes infiltrated in the auricular tissue. (**i**) Gene expression levels in the right auricle. Different letters indicate statistically significant differences (Tukey’s HSD method or Steel’s method, respectively).

Five-week-old male SD rats were purchased from Japan SLC Corporation (Shizuoka, Japan) and housed in cages at the Kobe Gakuin University Conventional Animal Experimental Breeding Room. The room maintained a light/dark cycle of 12 h each, a temperature of 22.5 ± 0.5 °C, and a humidity level of 55.0 ± 5.0%. The rats were acclimated for one week prior to the experiment, during which they were fed a sucrose-free general diet (CE-2; Nippon Crea Co., Ltd., Tokyo, Japan) and tap water. At the start of the experiment, the rats were randomly assigned to one of three groups: NC (n = 6), Water (n = 6), and Sucrose (n = 6). Rats in the NC group were defined as normal controls and were not sensitized or induced to have an allergy by DNFB. The rats in the NC and Water groups were fed CE-2 and tap water, while the rats in the Sucrose group were administered a solution of sucrose (Nacalai Tesque, Kyoto, Japan) dissolved in tap water at a concentration of 10% (*w*/*v*) for 16 d. On d 1 and 17 of the experiment, fecal samples were collected to examine changes in the composition of the intestinal microbiota and SCFA concentrations. To increase the concentration of blood acetate, a separate experiment was conducted. In this experiment, a Triacetin group (n = 6) was established alongside the NC, Water, and Sucrose groups. The Triacetin group was administered a 10% sucrose solution with 1% (*v*/*v*) triacetin (Nacalai Tesque). All other settings were consistent with the experiment depicted in Figure 1. Differences in the devices used for measurements in these separate experiments are detailed in the subsequent Methods section. Body weight and food and water intake were measured daily. This study was approved by the Kobe Gakuin University Animal Experimentation Committee (Animal Experimentation Certificate No. 21-17, 22-09, 23-05).

### 2.2. Experimental Model of Rat ACD

On the seventh day of the experiment, the backs of the rats were shaved. On d 8 and 9, 50 μL of DNFB (Nacalai Tesque) solution diluted to 0.5% was applied to the backs of the animals using an acetone–olive oil mixture (Nacalai Tesque) at a ratio of 3:1 to induce allergic sensitization. On d 15, a 0.2% DNFB solution was applied to the right auricle to induce an allergic reaction. The vehicle solution was administered to rats in the NC group instead of the DNFB solution during the sensitization and challenge for allergy induction. The auricular thickness was measured over time to assess inflammatory symptoms using manual or spring-loaded digital calipers. The use of different calipers may lead to variations in the measurements owing to differences in the applied pressure. To ensure consistency, all measurements with manual calipers were performed by the same operator with a standardized force, whereas spring-loaded calipers were used to automatically apply consistent pressure. These methodological differences were considered in the analyses. Dissection was performed on d 16 or 17. The cecal tissue was separated from the small intestine and colon, frozen in liquid nitrogen, and stored at −80 °C until SCFA analysis. The auricular tissues were immersion-fixed using 4% PFA, resin-embedded using Technovit®7100 (KULZER, Hanau, Germany), sectioned, and HE-stained.

### 2.3. Serum Uric Acid Quantification

The serum uric acid concentration was quantified using a uric acid C-test kit (Fujifilm Wako, Osaka, Japan) and determined from a standard curve derived from measurements of standard solutions with known concentrations.

### 2.4. Isolation of Total RNA, Reverse Transcription, and qPCR

Total RNA was extracted from the spleen, small intestine, or auricle collected at autopsy using Sepasol®-RNA I Super G (Nacalai Tesque) and the RNeasy Fibrous Tissue Mini Kit (QIAGEN, Venlo, The Netherlands). The genomic DNA was removed, and the RNA was reverse transcribed using the ReverTra Ace™ qPCR RT Master Mix with gDNA Remover (TOYOBO, Osaka, Japan). Gene expression levels were measured using the KAPA SYBR® FAST qPCR Master Mix (2×) Kit (KAPA BIOSYSTEMS, Wilmington, MA, USA) and StepOne™ Real-Time PCR systems (Thermo Fisher Scientific, Waltham, MA, USA). The primer sequences used are listed in Table 1. The analysis was conducted using the ΔΔCt method, with *Actb* or *Hprt* serving as internal standards.

### 2.5. Measurement of SCFAs

Fecal or cecal samples of approximately 100 mg were collected in 2.0 mL tubes to which 1 mL of deionized water was added. The tubes were stored overnight at −25 °C. After thawing, the samples were placed on a shaker at 4 °C for 30 min and centrifuged on a Himac CF15R (Hitachi, Tokyo, Japan) at 15,000× *g* rpm for 30 min at 4 °C. A sample of 500 µL of the supernatant was transferred to a separate tube with 50 µL of 5 mol/L hydrochloric acid (HCl; Fujifilm Wako Chemicals, Osaka, Japan) and 500 µL of 1 mmol/L 4-methylvaleric acid prepared in dehydrated anhydrous diethyl ether (Fujifilm Wako Chemicals). The solution was mixed and allowed to stand on ice for 5 min, followed by centrifugation at 15,000× *g* rpm for 5 min at 4 °C. The sample solution was prepared by transferring the supernatant to a 1.5 mL tube containing a trace amount of anhydrous sodium sulfate (Hayashi Pure Chemical). For the serum samples, 100 µL was transferred to a 1.5 mL tube, followed by the addition of 50 µL of 5 mol/L HCl and 100 µL of 1 mmol/L 4-methylvaleric acid. The mixture was then placed on ice for 5 min and centrifuged at 15,000× *g* rpm for 5 min at 4 °C. The sample solution was prepared by transferring the supernatant to a 1.5 mL tube containing a trace of anhydrous sodium sulfate. SCFA determination was carried out on a gas chromatography–mass spectrometry (GC-MS) instrument (GCMS-QP2010; Shimadzu, Kyoto, Japan) equipped with an HP-5 column (19091J-433; Agilent Technologies, Santa Clara, CA, USA), following the method described in [17]. A glass vial was used to combine 5 µL of *N*,*O*-Bis(trimethylsilyl)trifluoroacetamide (BSTFA; Nacalai Tesque) and 100 µL of the sample solution. The mixture was then incubated at 37 °C for 1 h. Next, 1 µL of the sample was subjected to GC-MS and measured. Quantification was performed using the internal standard method with a fixed amount of added 4-methylvaleric acid as the standard.

### 2.6. Measurement of Sodium in Fecal Samples

The fecal and cecal samples were freeze-dried and weighed. Then, 0.1 mol/L HCl, 9 times the sample volume, was added and the mixture was stirred vigorously at room temperature for 30 min. The supernatant obtained after centrifugation was used as the sample solution. The sample solution was diluted 100-fold with pure water and subjected to determination of the sodium and potassium contents using an atomic absorption spectrophotometer (ZA3000; Hitachi).

### 2.7. Microbiome Analysis

The QIAamp Fast DNA Stool Mini Kit (QIAGEN) was used to extract bacterial DNA from the fecal samples. The V3-V4 region of the 16S rRNA gene in the extracted fecal DNA was amplified using Illumina’s official protocol. The sequence of the forward primer (V3) was 341F (5′-CCTACGGGNGGCWGCAG-3′), and that of the reverse primer (V4) was 805R (5′-GACTACHVGGGTATCTAATCC-3′). Following preparation of the 16S rRNA amplicons, the resulting gene sequences were analyzed on a MiSeq next-generation sequencer. The amplicon sequences obtained were used to remove primer sequences, correct and remove PCR error sequences, and identify Amplicon Sequence Variants (ASVs) using QIIME2 and DADA2 (ver. 2021.2) [18,19]. Taxonomic analysis was performed using QIIME2 (Silva 138 99% OTUs full-length sequences) or BLAST.

### 2.8. Statistical Analysis

The data are presented as the means ± SEM. We used one-way ANOVA to test for the statistical significance of the means. If the *p* value was <0.05, Tukey’s multiple comparison test was used; otherwise, the Steel–Dwass multiple comparison test was used, and *p* < 0.05 was considered to indicate significant differences.

## 3. Results

### 3.1. Effects of Ingestion of 10% Sucrose Solution on Body Weight and Metabolic Parameters

We investigated how the ingestion of a 10% sucrose solution affects metabolic parameters, and the results are presented in Table 2. Intake of the sucrose solution for two weeks did not affect body weight gain but led to significant decreases in food intake and increases in water intake. Despite the reduced food intake, the consumption of liquid sucrose led to a significant increase in total energy intake in the Sucrose group compared to that in the control group. Liver weight and TG concentrations were measured to examine the effects of fructose absorption on the tissue due to sucrose intake. No significant differences were observed between the groups. Excess fructose uptake increases ATP consumption in the liver, resulting in the production of uric acid. The serum uric acid concentrations were significantly higher in the Water and Sucrose groups than in the control group. The uric acid concentration was higher in the Sucrose group than in the Water group, but the difference was not statistically significant.

### 3.2. Ingestion of 10% Sucrose Solution Accelerates Early Deterioration of DNFB-ACD Symptoms

In the Water group sensitized by DNFB and induced by applying DNFB solution to the auricle, the auricle became red and swollen (Figure 1a,b) and its thickness increased (Figure 1d) on d 2 after induction compared to the parameters in the control group, which was not induced by allergen stimulation. In contrast, the control group was not affected by the 10% sucrose solution. However, when rats that had consumed a 10% sucrose solution for two weeks were induced by the hapten, their auricular redness worsened compared to the condition in the Water group (Figure 1c). Additionally, their auricular thickness increased on the first day after induction compared to that in the Water group, and this increase was maintained until d 2 after induction (Figure 1d). These findings suggest that ingestion of a 10% sucrose solution may exacerbate the response to allergy induction. The number of inflammatory cells that infiltrated the auricular tissue was significantly higher in the Water group (Figure 1f) than in the control group (Figure 1e) and was highest in the Sucrose group (Figure 1g) compared to that in the other groups (Figure 1h).

DNFB-induced allergic reactions are delayed reactions with the involvement of T cells, especially Th1 cells, in the inflammation process [20]. We analyzed helper T cell subsets by examining the mRNA expression levels of genes that are highly expressed in each T cell type. The mRNA expression levels of *Gata3* (Th2), *Stat3* (Th17), and *Ikzf2* (Treg) were higher in the auricles of the Water and Sucrose groups, to which DNFB was applied, than in those of the control group, which was not subjected to DNFB stimulation (Figure 1i). In contrast, the expression level of *T-bet* (Th1) in the auricular tissues from the Water group was higher than that in the control and Sucrose groups, although the latter result did not reach statistical significance, and was significantly increased in the Sucrose group compared to the control group (Figure 1i). Moreover, the levels of the inflammatory cytokines *IL-1β* and *IL-18* produced by Th1 cells were higher in the Sucrose group than in the control group (Figure 1i). These findings show that consumption of a 10% sucrose solution for approximately two weeks promotes Th1-dominant inflammatory responses and exacerbates symptoms in DNFB-induced ACD. 

### 3.3. Ingestion of 10% Sucrose Solution Alters the Composition of the Intestinal Microbiota

**Figure 2:**
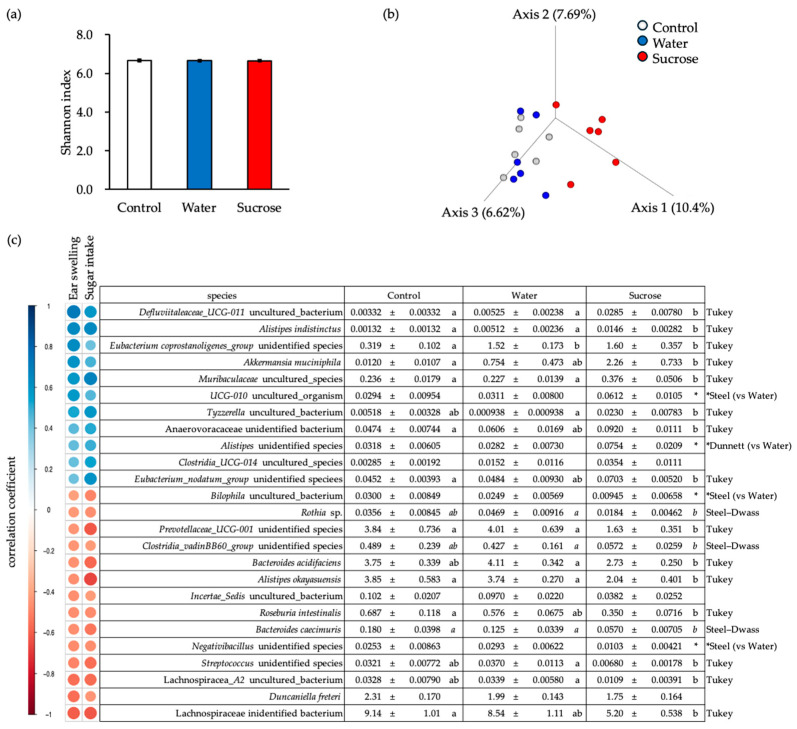
Ingestion of sucrose alters the composition of the intestinal microbiota. (**a**) Shannon index of gut microbiota in the control, Water, and Sucrose groups. (**b**) β-diversity analysis of gut microbiota in the three groups. (**c**) Pearson’s correlations of the bacterial species with the levels of ear swelling and sugar intake (left columns). Comparison and statistical analysis of the relative abundance of representative bacteria (right columns). Lowercase letters indicate significant differences between values. ‘*’ indicates a statistically significant difference from the Water group. The statistical methods used for each parameter are presented on the right-hand side of the table.

Sucrose intake affects the composition of the intestinal microbiota [13,21,22,23]. To investigate the effect of a 10% sucrose solution on the composition of gut microbiota, we analyzed bacterial DNA in feces and assessed the α- and β-diversity using amplicon sequence and read count data obtained via next-generation sequencing (Figure 2a). In the β-diversity analysis, a similar distribution was observed for each sample from the Water and NC groups. In contrast, each sample in the Sucrose group was distributed in a different region from that of the NC and Water groups (Figure 2b). This suggests that the intestinal microbiota composition differed between the Sucrose group and the NC and Water groups at the species level. We identified the bacterial species that contributed to the worsening of ACD symptoms after the ingestion of liquid sucrose. We included only the bacterial species that were present in more than 50% of the samples and correlated (|PEARSON coefficient| > 0.4) with auricular thickening on d 1 after allergy induction (Figure 1d). These species were also found to be positively correlated (|PEARSON coefficient| > 0.4) with sucrose intake.

The bacterial species with specifically increased fecal abundance ratios after sucrose intake compared to those in the Water group were *Defluviitaleaceae_UCG-011 uncultured_bacterium*, *Alistipes indistinctus* (a producer of SCFAs) [24], and *Akkermansia muciniphila* (SCFA producer) [25]. Auricular thickening was positively correlated with the abundance ratios of Muribaculaceae *uncultured_species* (a producer of SCFAs), *Tyzzerella uncultured_bacterium*, and *Alistipes* unidentified species. Conversely, compared to the Water group, sucrose consumption was specifically associated with reduced abundance ratios of *Bilophila uncultured_bacterium* and *Rothia* sp. in feces. The bacterial species identified in the samples were *Prevotellaceae_UCG-001* (unidentified species), *Clostridia_vadinBB60_group* (unidentified species), *Bacteroides acidifaciens*, *Alistipes okayasuensis*, *Bacteroides caecimuris*, *Streptococcus* (unidentified species), and *Lachnospiracea_A2* (uncultured bacterium). The abundance ratios of all bacterial species negatively correlated with auricular thickening. Unequivocal identification of bacterial species from the sequences of many amplicons obtained by next-generation sequencing was challenging. Comparisons of the nucleotide sequences revealed that Muribaculaceae *uncultured_species* could be identified as either *Duncaniella dubosii* strain H5 (99% identity) or *D. freteri* strain H5 (99% identity). Additionally, *D. freteri* strain TLL-A3 (98% identity) was identified based on an analysis of the nucleotide sequence [26]. Regarding *Tyzzerella*, the uncultured bacterium was closely related to *Anaerotignum lactatifermentans* strain G17 (97% identity) [27,28].

Studies have shown that SCFA-producing bacterial species are among the species positively correlated with sucrose intake, while negative correlations include fewer SCFA-producing bacteria. Our findings suggest that sucrose intake is positively associated with the abundance of bacterial species that produce SCFAs.

### 3.4. Blood Acetate and Butyrate Concentrations Decrease after Liquid Sucrose Intake

**Figure 3:**
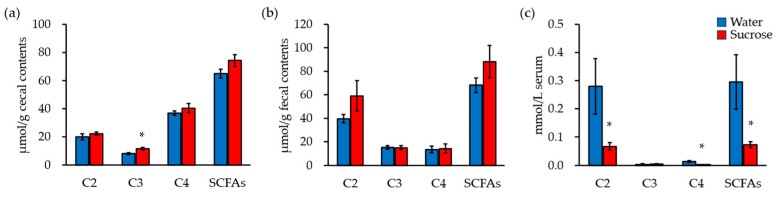
Blood acetate and butyrate concentrations decrease after sucrose intake. (**a**) The concentrations of acetate (C2), propionate (C3), butyrate (C4), and total SCFAs in the cecal contents. (**b**) The concentrations of C2, C3, C4, and total SCFAs in the fecal contents. (**c**) The concentrations of C2, C3, C4, and total SCFAs in the serum. ‘*’ indicates statistically significant differences (Student’s *t*-test).

Undigested food components are fermented by the gut microbiota to produce SCFAs and other organic acids. Ingestion of the sucrose solution altered the composition of the gut microbiota (Figure 2), potentially leading to modifications in the concentration of SCFAs in the gut lumen. To investigate whether any potential changes were present, we measured the SCFA concentrations in the cecal contents, feces, and serum. Analyzing short-chain fatty acids (SCFAs) separately in cecal contents and feces allows for the identification of differences in SCFA production kinetics, substrate utilization, and microbial activity at different anatomical locations within the colon. This approach provides valuable insights into the dynamic processes of microbial fermentation and SCFA production in the gastrointestinal tract. The cecal contents, representing an earlier stage of digestion and microbial fermentation, offer a glimpse into the initial breakdown of dietary fibers and other substrates by the gut microbiota. In contrast, fecal samples reflect the cumulative effects of microbial fermentation, absorption, and transit time in the colon. In the cecal contents, the concentrations of butyrate (C4), acetate (C2), and propionate (C3) were higher, in that order, and the concentration of propionate in the Sucrose group was significantly higher than that in the Water group (Figure 3a). In the feces, the concentrations of acetate, propionate, and butyrate were higher, in that order, with no significant differences between the two groups (Figure 3b). SCFAs in the intestinal lumen are absorbed by intestinal epithelial cells and transported to the portal vein. The serum concentrations of SCFAs were highest for acetate and lowest for butyrate and propionate, and the concentrations of acetate and butyrate were significantly lower in the Sucrose than in the Water group (Figure 3c). The results suggest that ingestion of a sucrose solution reduces SCFA absorption from the intestinal lumen, as indicated by the lower serum SCFA concentrations.

### 3.5. Sucrose Ingestion Promotes the Expression of Monosaccharide Transporters in the Small Intestine but Has No Effect on SCFA Transporters

**Figure 4:**
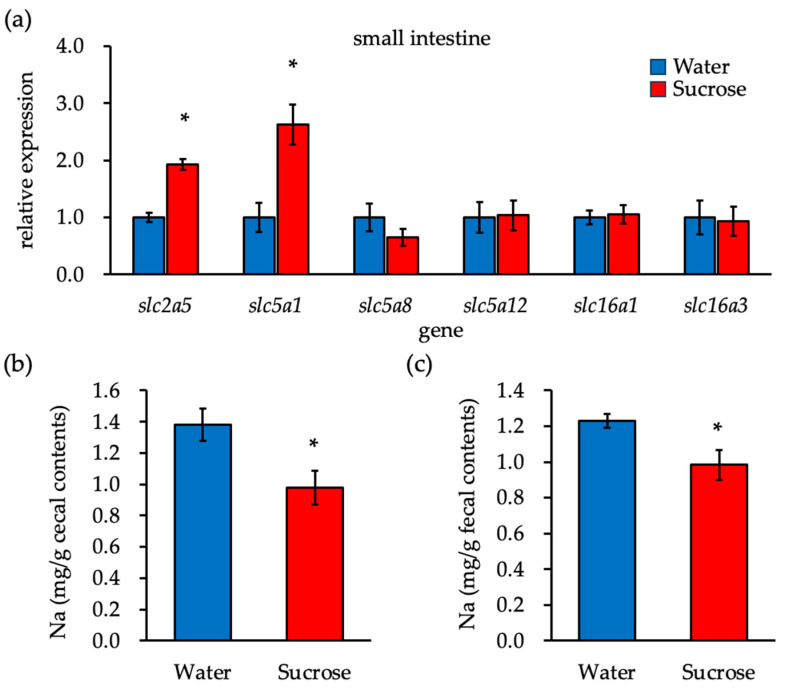
Sucrose intake promotes the expression of monosaccharide transporters, but not SCFA transporters, in the small intestine and reduces cecal and fecal sodium concentrations. (**a**) Gene expression levels in the small intestine. (**b**) The concentration of sodium in the cecal contents. (**c**) The concentration of sodium in the fecal contents. ‘*’ indicates statistically significant differences (Student’s *t*-test).

We measured the mRNA expression of SCFA and glucose and fructose transporters in the small intestine to investigate the cause of reduced SCFA absorption from the intestinal lumen after the ingestion of a sucrose solution. The expression levels of the glucose and fructose transporters *Slc2a5* (*GLUT5*) and *Slc5a1* (*SGLT1*) were significantly higher in the Sucrose group than in the Water group (Figure 4a). SGLT1 and SMCTs are sodium-dependent transporters. The expression of *Slc5a1* (*SGLT1*) increased significantly in the Sucrose group, indicating enhanced absorption of both glucose and sodium. The concentration of sodium in the cecal contents and feces decreased in the Sucrose group (Figure 4b,c). We conclude that the reduced SCFA absorption from the intestinal lumen after sucrose intake is caused by an increase in SGLT1 expression and sodium uptake in the small intestine. Additionally, the reduced concentration of sodium in the intestinal lumen can be attributed to the action of SMCTs. This is due to the decreased uptake of SCFAs by SMCTs, which antagonize SGLT1 for sodium uptake.

### 3.6. Triacetin Intake Reverses Early Exacerbation of DNFB-ACD Symptoms Associated with Sucrose Ingestion

**Figure 5:**
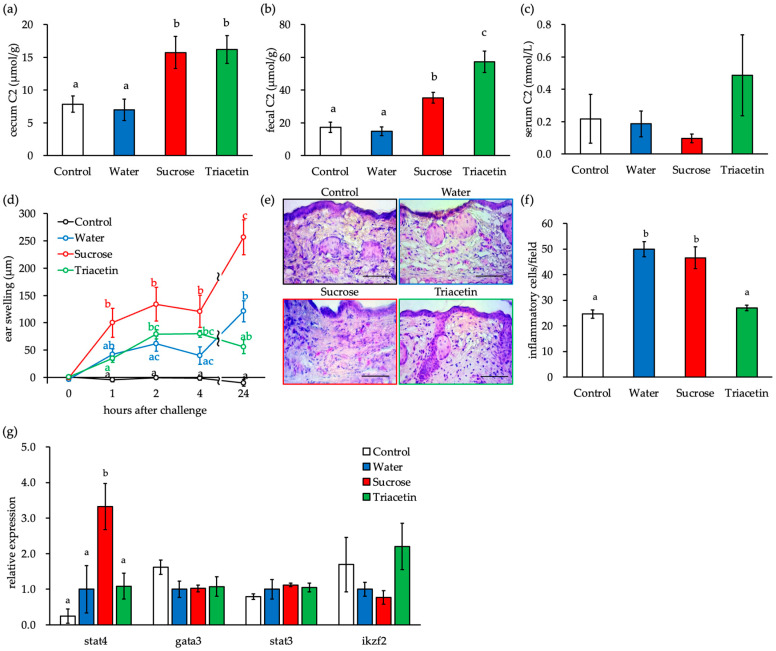
Triacetin intake reverses the early exacerbation of DNFB-ACD symptoms associated with sucrose ingestion. (**a**) The concentration of C2 in the cecal contents. (**b**) The concentration of C2 in the fecal contents. (**c**) The concentration of C2 in the serum. (**d**) Time course of thickening of the right auricle sensitized to DNFB. (**e**) HE staining of tissue sections of the right auricle. The scale bar indicates 50 µm. (**f**) Number of granulocytes infiltrated in the auricular tissue. (**g**) Gene expression level in the right auricle. Different letters indicate statistically significant differences (Tukey’s HSD method). Areas without any symbols indicate the absence of any significant differences.

Triacetin is a short-chain TG that is metabolized to acetate by lipases in the small intestinal lumen. To assess whether the decreased blood SCFA concentration was at least one of the causes for the worsening of ACD symptoms with the ingestion of liquid sucrose, we examined whether symptoms recovered when triacetin was added to the sucrose solution. The concentration of acetate in the cecal contents and feces of the Sucrose group increased significantly (Figure 5a,b), while the serum acetate concentrations were lower (Figure 5c) than those in the control and Water groups. The concentration of acetate did not differ in the cecal contents of the Sucrose and Triacetin groups, the latter of which consumed a mixture of sucrose solution and 1% triacetin (Figure 5a), but was significantly higher in the feces of the Triacetin group (Figure 5b). Additionally, the concentration of serum acetate was higher in the Triacetin group than in the other groups (Figure 5c). Auricular thickness was significantly higher in the Sucrose group induced by DNFB application than in the Water group at all examined time points (Figure 5d). Moreover, the infiltration of inflammatory cells in the auricular tissue 24 h after sensitization with DNFB was significantly higher in the Sucrose group than in the Water group (Figure 5e,f). We analyzed the expression levels of marker genes of helper T cell subsets in auricular tissues. The expression level of *Stat4*, a marker gene for Th1 cells, was higher in the Water group than in the control group and significantly higher in the Sucrose group than in the control and Water groups (Figure 5g). The ACD symptoms due to DNFB application in the Sucrose group, as described above, were restored to the levels of the Water group after the simultaneous intake of 1% triacetin (Figure 5d–g). These findings suggest that the ingestion of a sucrose solution may worsen ACD symptoms by decreasing the concentration of SCFAs in the blood.

## 4. Discussion

We investigated the effects of the ingestion of a sucrose solution on DNFB-induced ACD and their underlying mechanism. The results showed that the ingestion of a 10% sucrose solution for approximately two weeks exacerbated ACD symptoms caused by a single application of DNFB. This symptom worsening can be attributed, at least in part, to a decrease in blood SCFA levels. SCFAs affect the activity of various innate and adaptive immune cells and influence and suppress symptoms of allergic diseases [29,30]. Studies have reported low SCFA concentrations in the feces of patients with atopic dermatitis, and similar trends have been observed in animal models [31,32]. Moreover, subcutaneous injection of butyrate, a type of SCFA, suppresses contact hypersensitivity induced by 2,4,6-trinitro-1-chlorobenzene (TNCB) [33]. Although the effect of sucrose consumption on blood SCFA concentrations is currently unclear, studies have shown that the concentrations of acetate and butyrate in the blood are lower in mice fed lard, sucrose, and fructose than in those fed only lard and sucrose [34]. Acetate and butyrate inhibit the activity of histone deacetylases (HDACs), maintain a histone acetylation state, and induce Tregs that suppress other helper T cells [35]. Under our experimental conditions, a decrease in the number of Treg cells in the auricular tissue due to sucrose ingestion was not observed. The observed increase in the number of Th1 cells promoted by DNFB is likely attributed to the decreased Th1 suppression of Treg cells caused by the reduced blood SCFA concentration.

The ingestion of liquid sucrose resulted in changes in the composition of the intestinal microbiota and increased the concentrations of SCFAs in the intestinal lumen. The enrichment of SCFA-producing bacteria after sucrose intake suggests a correlation with the increased SCFA concentrations in the gut lumen. The decreased SCFA concentrations in the blood could be partially attributed to reduced SCFA absorption from the intestinal lumen. SCFAs exist in molecular form (R-COOH) and dissociated ionic form (R-COO^−^) in the gastrointestinal lumen. For instance, acetic acid can exist as CH_3_COOH and CH_3_COO^−^, with an acid dissociation constant (pKa) of 4.56. Considering the pH of the small and large intestinal lumen [36], it is believed that most acetic acid molecules exist in the dissociated state, CH_3_COO^−^. Uncharged SCFAs can diffuse into cells. Dissociated SCFAs, on the other hand, are negatively charged and cannot pass through cell membranes. Instead, they are taken up in the small and large intestines by the sodium ion-dependent monocarboxylate transporters SLC5A8/SMCT1 and SLC5A12/SMCT2, which are expressed on the apical surface of small intestinal absorptive cells and colonic epithelial cells. The absorbed SCFAs are thought to be transported into the blood by the monocarboxylate transporter SLC16a1/MCT1, which is expressed on the basement membrane of the absorptive cells [37]. Therefore, SCFA absorption from the intestinal lumen depends on the sodium ion-dependent transporters SMCT1 and SMCT2. Sucrose ingestion did not alter the expression levels of SCFA transporter genes in the small intestine. Therefore, the observed phenomenon cannot be attributed to the decreased expression of SCFA transporters.

Sucrose ingested by humans is broken down into glucose and fructose by sucrase, which is located on the apical plasma membrane of small intestinal absorptive cells. Glucose is actively transported into the small intestinal absorptive cells via the sodium ion-dependent transporter SLC5A1/SGLT1, while fructose is absorbed through facilitated diffusion via SLC2A5/GLUT5. Fructose is metabolized to fructose-1-phosphate by ketohexokinase in the absorbing cells and then converted to glucose and organic acids [38]. Sucrose or fructose, particularly in solution, reportedly increases the mRNA expression of sucrase, *SGLT1*, *GLUT5*, and *GLUT2* in the small intestinal epithelial cells [39,40]; however, this effect is not observed with glucose [39]. We hypothesize that the consumption of a 10% sucrose solution increases the expression of sucrase, SGLT1, GLUT5, and GLUT2 in the small intestine, leading to enhanced degradation of sucrose and uptake of glucose and fructose into the absorbing cells. The mRNA expression levels of *SGLT1* and *GLUT5* in the small intestine increased in the Sucrose group compared to the Water group. The results indicate that intake of a sucrose solution greatly enhances glucose and sodium ion uptake via SGLT1 due to the increased expression of SGLT1 in the small intestine; consequently, the concentration of sodium decreases in the cecal contents and feces. The ingestion of a sucrose solution may decrease SCFA uptake by the sodium-dependent SCFA transporter, leading to reduced SCFA levels in the blood due to the decrease in sodium concentration in the gut lumen.

Our study revealed that the ingestion of a 10% sucrose solution for two weeks disrupted the composition of the gut microbiota and worsened ACD symptoms by hindering SCFA absorption from the intestines. We propose that the consumption of liquid sucrose impairs SCFA absorption and that the effect is linked to a reduced sodium concentration in the gut lumen as a result of increased glucose and sodium uptake; the upregulation of SGLT1 expression in the small intestine facilitates this event. The administration of triacetin led to increased acetate concentrations in the blood and partly ameliorated ACD symptoms exacerbated by sucrose intake. This statement highlights a crucial mechanistic insight: excessive sucrose intake can exacerbate ACD due to diminished sodium-dependent absorption of SCFAs from the intestines, counteracting glucose uptake.

## 5. Conclusions

Regular intake of a sucrose solution exacerbated symptoms of ACD in rats, potentially mediated by alterations in the gut microbiota composition and reduced absorption of SCFAs due to the increased expression of sodium-glucose transporter 1 (SGLT1). These findings raise concerns about regular sucrose solution consumption potentially exacerbating allergic dermatitis.

## Figures and Tables

**Table 1:** Primer sequences used in this study.

Target Gene	Forward Primer Sequence (5′->3′)	Reverse Primer Sequence (5′->3′)
*actb*	GGAGATTACTGCCCTGGCTCCTA	GACTCATCGTACTCCTGCTTGCTG
*gata3*	GGCTACGGTGCAGAGGTATC	GATGGACGTCTTGGAGAAGG
*hprt*	CTCATGGACTGATTATGGACAGGAC	GCAGGTCAGCAAAGAACTTATAGCC
*ikzf2*	GGCTTCCGAATGGTAAACTG	CATTTGAACGGCTTCTCTCC
*slc16a1*	TCTTTGGATTTGCCTTTGGT	TGAGGCGGCCTAAAAGTG
*slc16a3*	CTACAGCGACACAGCTTGGA	GACCCCTGTGGTGAGGTAGA
*slc2a5*	AGAAGACAGGGAAGCTGACC	CTGCTGCATGAACTCTGAGG
*slc5a1*	CATGCCTAACGGACTTCGAG	CGAGGATGAACAACCTTCCT
*slc5a8*	TCAAGGTGGCATCAATACGA	TCCAGAACGTGTGTCTTTGC
*stat3*	AAGAGTCTCGCCTCCTCCAG	ATCTGCTGCTTCTCCGTCAC
*stat4*	GATCTGCCTCTATGGCCTCA	AGGAGTTGGCCCAAGGTAAC
*tbx21*	GGAACCGCTTATACGTCCAC	CTTATGGAGGGACTGCAGGA
*il-1* *β*	AGGAGAGACAAGCAACGACAA	GTTTGGGATCCACACTCTCCA
*il-18*	GACAAAAGAAACCCGCCTG	ACATCCTTCCATCCTTCACAG

**Table 2:** Effects of ingestion of 10% sucrose solution on body weight and metabolic parameters.

	Control	Water	Sucrose
initial body weight (g)	194 ± 4.78	198 ± 4.38	198 ± 5.89
total body weight gain (g)	107 ± 4.83	112 ± 2.62	113 ± 8.91
total food ingestion (g)	391 ± 12.6 a	406 ± 11.6 a	314 ± 20.3 b
total water ingestion (g)	586 ± 48.5 a	658 ± 87.6 a	1240 ± 118 b
total energy intake (kcal)	1140± 47.2 a	1390 ± 43.5 a	1570 ± 93.0 b
liver weight (g)	15.1± 0.858	15.2 ± 0.879	15.3 ± 1.31
liver triglyceride (mg/g)	24.0 ± 2.24	20.7 ± 1.18	20.5 ± 1.47
serum urate (mg/dL)	0.631 ± 0.119 a	1.94 ± 0.262 b	2.89 ± 0.407 b

Lowercase letters indicate significant differences between values. Tukey’s method was used to evaluate total food ingestion, total water ingestion, and serum urate levels; Steel’s method was used to evaluate total energy intake.

## Data Availability

The data presented in this study are available on request from the corresponding author due to an ongoing research project.
